# Persistent Fever and Skin Lesions Due to Histoplasmosis in a Boy from Rural Nepal

**DOI:** 10.4269/ajtmh.15-0664

**Published:** 2016-02-03

**Authors:** Shovit Thapa, Sunil Chandra Jha, Andrew B. Trotter

**Affiliations:** Department of Internal Medicine, Tribhuvan University Teaching Hospital, Kathmandu, Nepal; Department of Cardiology, Manmohan Cardiothoracic and Vascular Transplant Centre, Tribhuvan University Teaching Hospital, Kathmandu, Nepal; Department of Public Health and Community Medicine, Tufts University School of Medicine, Boston, Massachusetts

**Figure 1.**
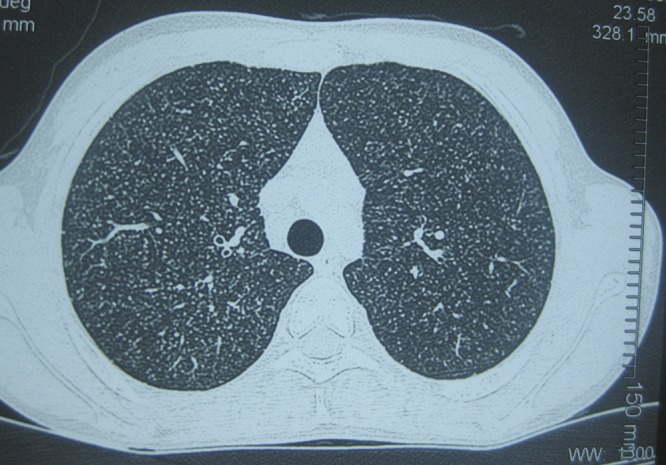
Computerized tomography of the chest after initial hospitalization showing a diffuse, bilateral, miliary pattern in the lung fields.

**Figure 2.**
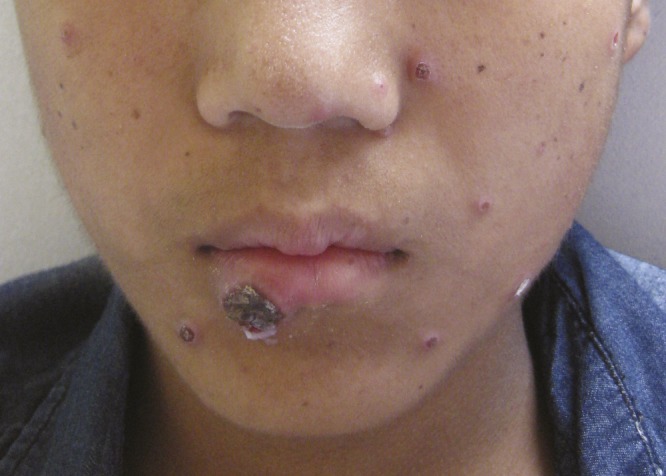
Multiple skin lesions on the face that were found during the second hospital admission.

**Figure 3.**
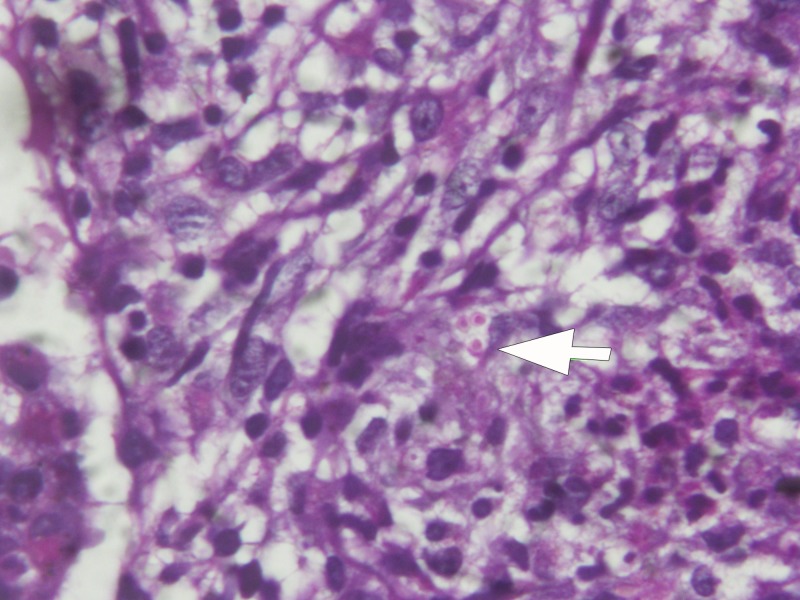
Skin biopsy of a representative skin lesion using periodic acid–Schiff staining reveals small organisms consistent with *Histoplasma* spp. Similar organisms were seen in the lung biopsy (not shown).

A 14-year-old Nepali male with a history of rheumatic heart disease complicated by severe mitral stenosis presented with a 2-week history of high-grade fever, dry cough, and hepatosplenomegaly on examination. He described chronic exposure to chickens but no other significant exposure history. Computerized tomography scan of the chest revealed a bilateral diffuse miliary pattern (Figure 1

). A transthoracic echocardiogram showed no change in his valvular disease from previous studies and multiple blood cultures were negative. He was diagnosed with miliary tuberculosis; started on isoniazid, rifampicin, ethambutol, and pyrazinamide; and discharged. After 3 weeks, he was readmitted for persistent high-grade fever, chills, dry cough, and a 7-kg weight loss. He also developed small papular lesions on his face that became ulcerative after steroids were introduced (Figure 2

). He underwent a video-assisted thoracoscopic lung biopsy, and skin biopsy, which showed small organisms consistent with *Histoplasma* spp. (Figure 3

). Initially, the patient was treated with oral itraconazole 200 mg three times daily for 3 days then twice daily, but by day 7 of treatment, he still continued to have persistent high-grade fever, cough, and chest pain. He was subsequently treated with amphotericin B deoxycholate with clinical improvement within 2 days of initiating therapy. He was treated with amphotericin B for 2 weeks and transitioned to oral itraconazole. Human immunodeficiency virus testing was negative.

Histoplasmosis has only been described twice in Nepal: once in an Indian national who traveled to Nepal for medical care^1^ and once in a Nepali in Nepal,^2^ making this the second case described in the literature in a Nepali citizen. Increased recognition of infectious diseases in areas where it was previously not thought to be prevalent and changing global distribution of infectious diseases can be an important clue to guide diagnostic workup and direct public health awareness. This case not only highlights the presence of histoplasmosis in Nepal but further supports a recent review describing increased recognition of histoplasmosis in other parts of Asia.^3^ In tuberculosis-endemic regions, disseminated histoplasmosis can easily be mistaken for tuberculosis owing to its similar clinical presentation and must be considered in patients who do not respond to empiric antitubercular therapy.
